# Nitric Oxide Deficiency in Mitochondrial Disorders: The Utility of Arginine and Citrulline

**DOI:** 10.3389/fnmol.2021.682780

**Published:** 2021-08-05

**Authors:** Mohammed Almannai, Ayman W. El-Hattab

**Affiliations:** ^1^Section of Medical Genetics, Children’s Hospital, King Fahad Medical City, Riyadh, Saudi Arabia; ^2^College of Medicine, King Saud University, Riyadh, Saudi Arabia; ^3^Department of Clinical Sciences, College of Medicine, University of Sharjah, Sharjah, United Arab Emirates; ^4^Clinical Genetics, University Hospital Sharjah, Sharjah, United Arab Emirates

**Keywords:** arginine, citrulline, nitric oxide, MELAS, mitochondria

## Abstract

Mitochondrial diseases represent a growing list of clinically heterogeneous disorders that are associated with dysfunctional mitochondria and multisystemic manifestations. In spite of a better understanding of the underlying pathophysiological basis of mitochondrial disorders, treatment options remain limited. Over the past two decades, there is growing evidence that patients with mitochondrial disorders have nitric oxide (NO) deficiency due to the limited availability of NO substrates, arginine and citrulline; decreased activity of nitric oxide synthase (NOS); and NO sequestration. Studies evaluating the use of arginine in patients with mitochondrial myopathy, encephalopathy, lactic acidosis, and stroke-like episodes (MELAS) presenting with stroke-like episodes showed symptomatic improvement after acute administration as well as a reduction in the frequency and severity of stroke-like episodes following chronic use. Citrulline, another NO precursor, was shown through stable isotope studies to result in a greater increase in NO synthesis. Recent studies showed a positive response of arginine and citrulline in other mitochondrial disorders besides MELAS. Randomized-controlled studies with a larger number of patients are warranted to better understand the role of NO deficiency in mitochondrial disorders and the efficacy of NO precursors as treatment modalities in these disorders.

## Introduction

Primary mitochondrial disorders are clinically heterogeneous disorders that result from mitochondrial dysfunction caused by inherited or *de novo* defects in mitochondrial DNA (mtDNA) or nuclear encoded mitochondrial genes. Mitochondria are important for cellular energy production through oxidative phosphorylation (OXPHOS) and the electron transport chain. Energy failure contributes to multi-organ involvement usually seen in mitochondrial disorders. Mitochondria also perform other biological functions, including calcium homeostasis, steroid synthesis, and apoptosis regulation. Therefore, the underlying pathophysiology in mitochondrial disorders is complex and not completely understood (Dyakova et al., 2015; Gorman et al., 2016).

While recent advances in diagnostic technologies have expanded the list of mitochondrial disorders that we know, therapeutic interventions including symptom specific therapies and supportive measures remain limited. There are several ongoing preclinical and clinical trials that aim to develop more specific and effective treatment options for mitochondrial disorders (Pitceathly et al., 2021).

There is growing evidence that patients with mitochondrial diseases have nitric oxide (NO) deficiency, and this can contribute to some of the complications observed in these patients. The best understood example is stroke-like episodes in patients with mitochondrial myopathy, encephalopathy, lactic acidosis, and stroke-like episodes (MELAS) (Koga et al., 2012). Given the myriad roles of NO as a signaling molecule, better understanding of the mechanisms of NO deficiency in mitochondrial disorders and how this deficiency can contribute to various disease manifestations will allow us to better treat these disorders.

In this article, we will review the current known evidence for NO deficiency and its pathophysiology in mitochondrial diseases and the possible therapeutic interventions.

## Synthesis and Biological Functions of Nitric Oxide

Furchgott and Zawadzki (1980), show that the removal of the endothelium resulted in loss of the action of acetylcholine as a vasodilator. The endothelium related agent responsible for relaxation of the vascular smooth muscles was not known at that time and was called endothelium-dependent relaxing factor (EDRF). Seven years later, this EDRF was shown to be NO (Ignarro et al., 1987; Palmer et al., 1987). NO, a colorless gas, is one of the oxides of nitrogen (Bruckdorfer, 2005). It is synthesized through the action of NO synthase (NOS) (Förstermann and Sessa, 2012). There are three isoforms of this enzyme; neuronal (nNOS), inducible (iNOS), and endothelial (eNOS). NOS uses arginine as a substrate for NO production. The catalytic reaction involves molecular oxygen and NADPH (nicotinamide adenine dinucleotide phosphate) (Mungrue et al., 2003). Citrulline is a by-product of the reaction. While intracellular arginine concentrations exceed the amount required as a substrate for NOS, acute introduction of exogenous arginine does increase NO production “arginine paradox” (Dioguardi, 2011).

**FIGURE 1:**
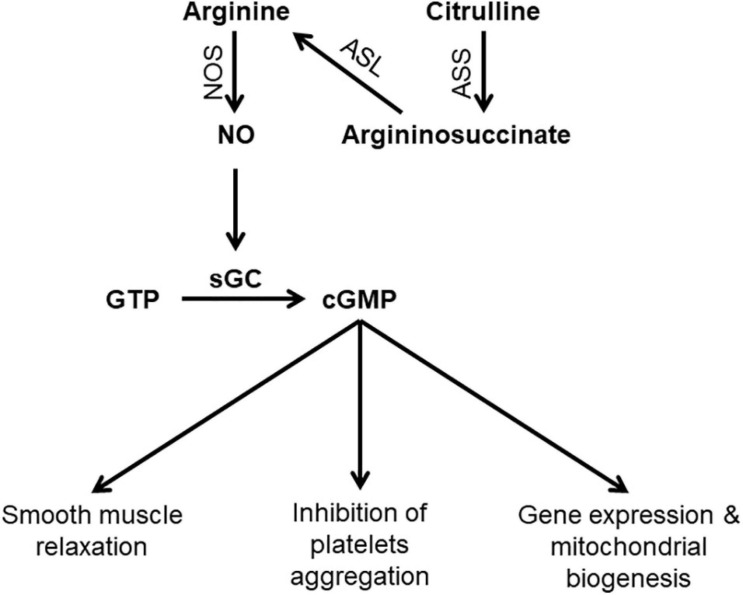
Schematic diagram showing steps in NO synthesis and potential consequences of NO deficiency. ASL, argininosuccinate lyase; ASS, argininosuccinate synthase; cGMP, Cyclic guanosine monophosphate; GTP, Guanosine-5′-triphosphate; sGC, Soluble guanylyl cyclas; NO, nitric oxide; NOS, nitric oxide synthase.

Arginine is derived via dietary protein intake, protein breakdown, and *de novo* synthesis (Keshet and Erez, 2018). The latter contributes to around 10–15% of whole body arginine (Luiking et al., 2012). Arginine is synthesized from citrulline through the sequential action of two enzymes; argininosuccinate synthase (ASS) and argininosuccinate lyase (ASL) (Figure 1). Citrulline itself is synthesized in the small intestine and is then transported to the kidney where it is converted to arginine in what is called “intestinal-renal axis” (Curis et al., 2005). Therefore, both arginine and citrulline are precursors for NO synthesis.

NO has diverse physiological functions including vasodilation, neurotransmission, and non-specific immunity. The presence of different isoforms of NOS allows NO production in response to various stimuli to carry on isoform specific functions (Bruckdorfer, 2005). NO is the most potent endogenous vasodilator. Shear stress induces eNOS activation resulting in increased NO production which activates the enzyme soluble guanylate cyclase (sGC), the primary receptor for NO. Activation of sGC produces cyclic guanosine monophosphate (cGMP), which is the second messenger system that mediates the cellular effects of NO including vasodilation and inhibition of platelet aggregation (Gewaltig and Kojda, 2002). The interaction of NO and sGC is important and dysregulation of this pathway has been linked to a variety of diseases such as heart disease and neurodegeneration (Sürmeli et al., 2015).

NO is also a neurotransmitter that is released at both, pre- and postsynaptic endings. At presynaptic endings, NO works as an anterograde neurotransmitter and it plays important roles in controlling gastrointestinal motility and pain transmission in the spinal cord (Picón-Pagès et al., 2019). In most regions of the brain, NO is released postsynaptically as a retrograde neurotransmitter. NO in turn stimulates glutamate exocytosis from the presynaptic endings and maintains what is called long term potentiation, which in turn is essential for learning and memory (Bon and Garthwaite, 2003).

## NO Deficiency in Mitochondrial Disorders; Underlying Mechanisms and Pathophysiology

### Mechanisms of NO Deficiency in Mitochondrial Disorders

In the last two decades, there is growing evidence that NO deficiency develops in patients with mitochondrial diseases and contributes to the complex pathophysiology of these disorders. In patients with MELAS, Koga et al. (2005) showed that NO metabolite (nitrate and nitrites) concentrations were lower compared to controls during the acute phase. Stable isotope infusion techniques used to evaluate NO synthesis in adult and pediatric subjects with MELAS (El-Hattab et al., 2016, 2012) showed lower NO production in affected subjects compared to controls, providing additional evidence of NO deficiency in mitochondrial disorders.

The mechanism of NO deficiency in patients with mitochondrial disorders is complex and results from several factors that act at different levels of NO synthesis. As mentioned earlier, both arginine and citrulline are substrates for NO synthesis, therefore their deficiencies can negatively impact NO synthesis. Koga et al. (2005) showed that plasma concentrations of arginine and citrulline were lower in patients with MELAS compared to control subjects and this observation was seen in both acute and interictal phases (*p* = 0.01). Moreover, arginine levels in the acute phase were significantly lower than in the interictal phase (Koga et al., 2005). Arginine clearance was found to be higher in subjects with MELAS syndrome and this might contribute to the observed low levels (El-Hattab et al., 2012). As arginine is synthesized *de novo* from citrulline, low citrulline levels can also explain the low arginine levels. In fact, *de novo* arginine synthesis was shown to be lower in subjects with MELAS syndrome (El-Hattab et al., 2012). Hypocitrullinemia was documented in patients with MELAS and other mitochondrial disorders (Parfait et al., 1999; Naini et al., 2005). Citrulline is synthesized *de novo* by mitochondrial enzymes and therefore mitochondrial dysfunction was speculated to be the underlying reason for hypocitrullinemia (Parfait et al., 1999; Naini et al., 2005). In a more recent study, Al Jasmi et al. (2020) showed that patients with mitochondrial disorders other than MELAS had low plasma arginine and citrulline levels compared to controls.

Besides deficiency of NO precursors, reduced activity of NOS could also contribute to low NO levels in patients with mitochondrial disorders. In hybrid cell-lines, Desquiret-Dumas et al. (2012) showed that the m.3243A > G mutation in *MT-TL1* gene, the most common mutation in MELAS, resulted in a significant decrease in the nitrite-nitrate concentration indicating reduced NOS activity. Through quantification of NADPH diaphorase histochemistry, Rodrigues et al. (2016) performed a quantitative analysis of NOS activity in muscle fibers from patients with variable mitochondrial diseases. They showed that muscle fibers with reduced cytochrome-c-oxidase (COX) activity have low sarcoplasmic NOS activity (Rodrigues et al., 2016). In addition, a high NADH/NAD^+^ ratio that results from defective mitochondrial respiration also inhibits NOS (Koga et al., 2012).

Mitochondrial proliferation is a compensatory mechanism that develops in mitochondrial disorders and results in increased COX activity. As NO has a strong affinity for COX (Vos et al., 2001), this increase in COX activity results in more NO binding, NO sequestration, and decreased local availability (Naini et al., 2005). Interestingly, while the ragged-red fibers (RRFs) in mtDNA disorders that are associated with mitochondrial proliferation are COX negative; in MELAS, the RRFs are typically COX positive and this is called “MELAS paradox” (Naini et al., 2005). The elevated amount of COX in MELAS blood vessels in turn results in NO sequestration.

In mitochondrial disorders, oxidative stress results in elevated levels of asymmetric dimethylarginine (ADMA) as well as overproduction of reactive oxygen species (ROS); both of which impair NOS activity (El-Hattab et al., 2012). Finally, reduced NOS activity can result from down regulation of NOS to compensate for impaired cellular respiration in mitochondrial disorders (Tengan et al., 2007).

### Impact of NO Deficiency on Mitochondrial Disorders

MELAS is one of the most common mitochondrial disorders caused by mutations in mtDNA (Sproule and Kaufmann, 2008). Most data on NO deficiency in mitochondrial disorders are derived from studies on this particular disease. One of the hallmarks of MELAS is the development of stroke-like episodes, in which the ischemic regions do not correspond to the typical vascular territories seen in classic thrombotic or embolic strokes (Koenig et al., 2016). Stroke-like episodes are an important cause of morbidity and mortality in MELAS subjects. In MELAS, NO deficiency results in vasoconstriction leading to ischemia and hypoxemia (Koga et al., 2012). Flow-mediated vasodilation (FMD) was found to be lower in subjects with MELAS compared to controls (Koga et al., 2006). Neuropathological findings in patients with MELAS include ischemic-like lesions characterized by neuronal loss, capillary proliferation, and peri-lesional astrogliosis. These findings suggest that vascular dysfunction is an important factor in the complex pathophysiology of MELAS (Betts et al., 2006). Other disease manifestations seen in MELAS and other mitochondrial disorders are fatigue and exercise intolerance. It is estimated that 20% of patients with mitochondrial disorders experience such symptoms (Mancuso et al., 2012). NO enhances exercise performance and skeletal muscle function through various signaling pathways (Dyakova et al., 2015). In fact, NO triggers mitochondrial biogenesis through the activation of different signaling pathways, including the peroxisome proliferator-activated receptor-γ coactivator 1α (PGC-1α) axis (Lira et al., 2010; Bishop et al., 2018). In mice, ablation of eNOS resulted in impaired exercise performance and increased plasma lactate levels (Lee-Young et al., 2010). Therefore tackling NO deficiency could help in ameliorating fatigue and exercise intolerance.

## Arginine and Citrulline in Mitochondrial Disorders

As evidence is growing for the role of NO deficiency in the pathophysiology of mitochondrial disorders, augmentation of NO synthesis through the use of NO precursors is a potential therapeutic option. Initial studies were carried out on subjects with MELAS, as stroke-like episodes in this disorder represent a good example of the role of NO deficiency in the pathogenesis of this complication.

### Arginine Supplementation in the Acute Phase of Metabolic Strokes in MELAS

Koga et al. (2002) reported three patients with MELAS (age 15–18 years) who received either arginine (0.5 g/kg) or placebo within 1 h of the onset of stroke-like symptoms. Using ^99m^Tc-ECD single-photon emission computed tomography (SPECT), the authors showed that arginine administration resulted in improved microcirculation. The symptoms associated with stroke-like episodes also improved with arginine treatment (Koga et al., 2002). Following this study, a larger trial was conducted on 24 patients with MELAS with a total of 34 stroke-like episodes (Koga et al., 2005). As compared to placebo, arginine administration was associated with significant clinical and biochemical improvement following stroke-like episodes. Acute administration of arginine during stroke-like episodes resulted in the resolution of the transient changes observed in brain magnetic resonance imaging (MRI) within 1 week (Kitamura et al., 2016). Koga et al. (2018) also evaluated the efficacy of intravenous arginine administered within 6 h of stroke-like episodes in 10 patients with MELAS. The primary endpoint was the improvement in rates of headache and nausea/vomiting at 2 h after completion of the initial intravenous administration. Although there was improvement in symptoms, this outcome measure did not reach the study target of 30% improvement in 2 h. The authors speculated that extending the window for intravenous arginine administration from 3 h in their previous study to 6 h in this study may partly explain these findings (Koga et al., 2018; Ikawa et al., 2020; Table 1).

**TABLE 1:** Summary of previous studies evaluating the use of Nitric Oxide precursors in patients with mitochondrial disorders.

**Authors**	**Study population**	**Intervention**	**Outcome**
Koga et al. (2002)	Three patients with MELAS with a total of 16 stroke-like episodes	IV arginine (0.5 g/kg) or placebo administrated within 1 h of onset of stroke-like symptoms	Stroke-like episodes associated symptoms responded to arginine treatment Improved microcirculation as measured by SPECT
Koga et al. (2005)	24 patients with MELAS with total of 34 stroke-like episodes and 72 control subjects	IV arginine (0.5 g/kg) or placebo administrated within 1 h of onset of stroke-like symptoms Six patients were treated by oral administration of oral arginine (0.15–0.3 g/kg/d for 18 months) to prevent stroke-like episodes	All symptoms suggesting stroke dramatically improved Concentrations of lactate and pyruvate, L-arginine, L-citrulline, NO, cGMP, and ADMA returned to interictal-phase concentrations within 24 h After oral arginine supplementation, the frequency and severity of symptoms caused by the stroke decreased dramatically (*p* < 0.05)
Kitamura et al. (2016)	Two patients with MELAS	IV arginine infusion at acute phase of stroke-like episodes	Resolution of the transient changes observed in brain MRI within 1 week
Koga et al. (2018)	15 patients with MELAS	IV arginine administered within six 6 h of stroke-like episodes in 10 patients Oral administration of arginine at a dose of 0.3–0.5 g/kg/day orally in three divided doses for 2 years to 13 patients	The statistical hypothesis that “the rate of improvement in headache and nausea/vomiting at 2 h after completion of the initial intravenous administration is greater than 30%” was not achieved. Nevertheless, the improvement rates increased with time Chronic arginine administration was not associated with a statistically significant difference in MELAS stroke scale, mitochondrial disease severity scores, or migraine severity score The interictal phases were extended after arginine treatment The maximum plasma arginine concentration was 167 μmol/L when seizures developed
Koga et al. (2006)	15 patients with MELAS and 20 controls	IV arginine (0.5 g/kg)or placebo 0.15–0.3 g/kg/day oral arginine for 24 months.	Two hours after arginine loading, there was no improvement in FMD values in the controls but the values in MELAS patients improved (*p* < 0.05) FMD values after 2 years of arginine supplementation improved compared to the original values (*p* < 0.05) and returned to control levels in all MELAS patients
El-Hattab et al. (2012)	10 adults subjects with MELAS and 10 control subjects	Oral arginine (10 g/m^2^ body surface area/day divided every 4 h for 48 h). Oral citrulline (10 g/m^2^ body surface area/day divided every 4 h for 48 h).	Increase in plasma arginine and citrulline concentration, increased NO synthesis, increased *de novo* arginine synthesis (*P* < 0.05) Citrulline supplementation at the same dose resulted in greater increase in NO synthesis
El-Hattab et al. (2016)	Five children subjects with MELAS and Five control subjects	Oral arginine (10 g/m^2^ body surface area/day divided every 4 h for 48 h). Oral citrulline (10 g/m^2^ body surface area/day divided every 4 h for 48 h).	NO production rate increased with arginine (*p* < 0.05) and to a greater extent with citrulline (*p* < 0.001) supplementations Plasma arginine concentration increased with arginine (*p* < 0.001) and citrulline (*p* < 0.001) supplementations Plasma citrulline concentration did not change with arginine but increased with citrulline (*p* < 0.05) supplementation
Rodan et al. (2020)	Three siblings with MELAS and four healthy controls	Single dose of oral arginine (100 mg/kg) 6 week oral arginine (100 mg/kg three times daily)	CVR in MELAS subjects after single dose and 6-week arginine course was not significantly different, but there was a trend towards increasing CVR in the frontal cortex and a decrease in the occipital cortex A 29–37% reduction in baseline CBF in one patient following 6 weeks of arginine with a marked increase in functional MRI activation in response to visual cortex toward control values following a single dose and 6 weeks of arginine in the same patient
Al Jasmi et al. (2020)	Nine individuals with variable mitochondrial diseases, less than 18 years of age	Arginine or citrulline (500 mg/kg/day if weight is < 20 kg and 10 g/m^2^/day if weight is ≥ 20 kg) for 2 weeks followed by 2 weeks washout period and then subjects were crossed over to the other intervention	Average RHI increased 15% and 19% with arginine and citrulline supplementation, respectively

*CBF, cerebral blood flow; CVR, cerebrovascular reactivity; FMD, flow-mediated vasodilation; RHI, reactive hyperemic index; SPECT, single-photon emission computed tomography.*

In a consensus statement from the mitochondrial medicine society (MMS) regarding patient care standards for primary mitochondrial disease that was based on Delphi-consensus, it was stated that “IV arginine hydrochloride should be administered urgently in the acute setting of a stroke-like episode associated with the MELAS m.3243 A > G mutation in the *MT-TL1* gene and considered in a stroke-like episode associated with other primary mitochondrial cytopathies as other etiologies are being excluded. Patients should be reassessed after 3 days of continuous IV therapy” (Parikh et al., 2017). In a review regarding the recommendations for the use of arginine in the management of acute stroke-like episodes in patients with MELAS, it was recommended that a bolus of 0.5 g/kg continues infusion over 24 h of arginine should be given within 3 h of symptoms onset and continued for 3–5 days (Koenig et al., 2016). A recent, Delphi method based consensus statements for the management of mitochondrial stroke-like episodes from Europe stated that the use arginine is controversial as there is little evidence to support it (Ng et al., 2019). Based on the published studies we reviewed, the MMS consensus statement (Parikh et al., 2017), opinions of experts in this field (Koenig et al., 2016), and the authors personal experience, there are clear benefits for the use of arginine in MELAS syndrome. Therefore, it is recommended to treat patients with MELAS with intravenous arginine during stroke-like episodes.

### The Role of Chronic Supplementation of Arginine and Citrulline in MELAS

Six patients with MELAS received oral arginine (0.15–0.3 g/kg/day) for 18 months. This resulted in significant improvement in the frequency and severity of stroke-like episodes after treatment (Koga et al., 2005). In a multicenter, prospective, clinical trial, oral administration of arginine at a dose of 0.3–0.5 g/kg/day in three divided doses for the duration of 2 years in 13 patients with MELAS was not associated with a statistically significant difference in the study outcomes including MELAS stroke scale, mitochondrial disease severity scores, or migraine severity scores (Koga et al., 2018). Nevertheless, there was a tendency for arginine to improve symptoms as there was a trend toward improvement (*p* = 0.055) on the MELAS stroke scale. In addition, the interictal phases were extended after arginine treatment. The maximum plasma arginine concentration was 167 μmol/L when seizures developed and the authors suggested that maintaining plasma arginine levels ≥ 168 μmol/L may therefore prevent seizures (Koga et al., 2018). Arginine administration was also associated with an improvement in the endothelial function in patients with MELAS as measured by FMD (Koga et al., 2006).

Oral arginine administration to 10 adults with MELAS at a dose of 10 g/m^2^ body surface area divided every 4 h for 48 h resulted in an increased NO synthesis rate as measured by arginine-to-citrulline flux which represents NO synthesis rate (El-Hattab et al., 2012). The increase in NO synthesis was associated with a concomitant increase in *de novo* arginine synthesis and plasma arginine concentration. Citrulline supplementation at the same dose resulted in higher *de novo* arginine synthesis and greater increase in NO synthesis, suggesting that citrulline is a better precursor for NO as compared to arginine (El-Hattab et al., 2012). Similar results were also observed when the same study was performed on five children with MELAS (El-Hattab et al., 2016). Based on these findings, an open-label dose-finding and safety clinical trial will soon start to establish the maximum tolerated dose of citrulline in individuals with MELAS syndrome by measuring the incidence of dose-limiting toxicities. The study will also evaluate changes in cerebral blood flow and cerebrovascular reactivity by using arterial spin-labeling (ASL) magnetic resonance imaging (MRI) as a secondary outcome measure.^1^

Three siblings with MELAS were studied with ASL MRI to evaluate regional cerebral blood flow (CBF) and arterial cerebrovascular reactivity (CVR). CVR was measured using changes in blood oxygen level dependent (BOLD) signal in combination with changes in end tidal PCO2. CVR reflects the capacity of blood vessels to dilate in response to vasodilatory stimulus and it is therefore a marker for brain vascular reserve. Compared to controls, subjects with MELAS were found to have decreased CVR (*p* ≤ 0.002) and increased CBF (*p* < 0.0026) and this difference correlated with disease severity and percentage of mutant mtDNA (Rodan et al., 2015). CVR was inversely proportional to the increase in cerebral blood flow. The authors speculated that increased CBF may result from adaptive response to compensate for energy deficiency or it may represent a passive response to tissue acidosis. Endothelial dysfunction that results from abnormal mitochondria may also cause functional impairment of vasodilation in response to stimuli, thereby reducing CVR and increasing blood flow (Rodan et al., 2015). Following this observation, the authors designed a pilot study to assess the response of CBF and CVR to arginine supplementation in three siblings with MELAS (aged 17, 21 and 22 years) compared to four healthy controls. Visual cortex response in MELAS and controls were evaluated using functional MRI with a stimulus consisting of an alternating black and white checkerboard (Rodan et al., 2020). Subjects with MELAS received a single oral dose of arginine at a dose of 100 mg/kg and a CVR study was performed 1 h later. Two weeks later, subjects with MELAS were started on a 6-week course of oral arginine at 100 mg/kg divided three times daily and CVR study was repeated after the course. CVR in MELAS subjects after a single dose and a 6-week arginine course was not significantly different than baseline, but there was a trend toward increasing CVR in the frontal cortex and a decrease in the occipital cortex. Following 6 weeks of arginine, one patient had a 29% reduction in baseline CBF in the frontal cortex and a 37% reduction in CBF in the occipital cortex. In this patient, pre-treatment functional MRI activation in response to visual cortex stimulus was significantly decreased compared to controls and showed marked increase toward control values following a single dose and a 6-week arginine course (Rodan et al., 2020).

In a consensus statement from the MMS regarding patient care standards for primary mitochondrial disease, it was stated that “The use of daily oral arginine supplementation to prevent strokes should be considered in MELAS syndrome” (Parikh et al., 2017). Following the first stroke in patients with MELAS, it is recommended to start oral arginine at a daily dose of 0.15–0.30 g/kg in three divided doses (Koenig et al., 2016).

### Potential Benefits of Arginine and Citrulline Supplementation in Other Mitochondrial Disorders

While initial studies focused on the use of arginine and citrulline in MELAS, mitochondrial disorders share common pathophysiological mechanisms and long term complications. In fact, stroke-like episodes could develop in mitochondrial disorders other than MELAS (Barca et al., 2020). In a retrospective study that evaluated the use of intravenous arginine therapy for acute metabolic strokes in pediatric patients with mitochondrial diseases other than MELAS, nine out of 71 subjects received acute arginine treatment for one or more stroke-like episodes (total 17 episodes for the nine subjects) during the study duration of 8 years (Ganetzky and Falk, 2018). These nine patients include four with mtDNA pathogenic point mutations, one with mtDNA deletion, and the remaining four had nuclear gene related disorders (*FBXL4*, *POLG*, *NDUFS8*, and *SURF1)*. A positive clinical response to intravenous arginine occurred in 47% of stroke-like episodes. Additional 6% of episodes showed clinical benefit from multiple simultaneous treatments that included arginine. The presence of unilateral symptoms strongly predicted arginine response. There was also a trend toward increased arginine responsiveness in patients with mtDNA mutations compared to those with nuclear genes mutations (*P* = 0.1) and in older pediatric subjects compared to younger subject (*P* = 0.24).

In a recent study, nine individuals with variable mitochondrial diseases, less than 18 years of age, were randomized to receive either arginine or citrulline (500 mg/kg/day if weight is < 20 kg and 10 g/m^2^/day if weight is ≥ 20 kg; divided in three doses) for 2 weeks. The subjects then crossed over to the other intervention following a 2 week washout period (Al Jasmi et al., 2020). Peripheral arterial tonometry was used in this study to measure the reactive hyperemic index (RHI) as a marker for endothelial dysfunction. RHI averages increased after arginine or citrulline supplementation. Average RHI increased 15 and 19% with arginine and citrulline supplementation, respectively (Al Jasmi et al., 2020).

## Conclusion

In this review, we have presented published evidence of NO deficiency in patients with mitochondrial disorders. Furthermore, we have reviewed different mechanisms leading to NO deficiency in mitochondrial disorders and how this likely leads to the various complications observed in these disorders. Results from studies that evaluated the effect of arginine and citrulline supplementation in patients with mitochondrial disorders, in particular the effect on stroke-like episodes in patients with MELAS are presented and discussed.

Additional studies are warranted to better understand how NO deficiency contributes to the pathogenesis of complications seen in mitochondrial diseases and whether this is mediated by NO itself or downstream molecules. In fact, targeting the downstream pathways is another potential alternative that is being explored in current trials. As many of the physiological functions of NO are mediated through its primary receptor, soluble guanylyl cyclase (SGC), a phase 2a study to assess safety and tolerability of a CNS penetrant-SGC stimulator (IW-6463) in patients with MELAS is currently recruiting.^2^

Augmenting NO synthesis, through arginine or citrulline supplementation, showed clear benefits in patients with MELAS presenting with stroke-like episodes in different studies and therefore it is recommended to treat these patients with intravenous arginine 0.5 g/kg continues infusion over 24 h given within 3 h of symptoms onset and continued for 3–5 days followed a daily dose of arginine at 0.15–0.30 g/kg/day three times daily. As citrulline was shown to be superior to arginine as precursor for NO in small studies, future trials are warranted to validate this approach, identify a safe dosage regimen, and recognize subsets of patients who might benefit the most.

## Author Contributions

MA drafted manuscript. AE-H revised the manuscript. Both authors approved the final version of the manuscript and agreed to be accountable for all aspects of the work to ensure that questions related to the accuracy or integrity of any part of the work are appropriately investigated and resolved.

## Conflict of Interest

The authors declare that the research was conducted in the absence of any commercial or financial relationships that could be construed as a potential conflict of interest.

## Publisher’s Note

All claims expressed in this article are solely those of the authors and do not necessarily represent those of their affiliated organizations, or those of the publisher, the editors and the reviewers. Any product that may be evaluated in this article, or claim that may be made by its manufacturer, is not guaranteed or endorsed by the publisher.

## Footnotes

1
https://clinicaltrials.gov/ct2/show/NCT03952234

2
https://clinicaltrials.gov/ct2/show/NCT04475549
